# Cultural influences on coping among Chinese children aged 7–14 undergoing chemotherapy: implications for pediatric oncology service delivery

**DOI:** 10.1186/s12913-026-14733-6

**Published:** 2026-05-23

**Authors:** Shishuang Zhou, Huiyuan Li, Haiying Huang, Xiao Pin, Mimi Wai Man Chan, Yuan Yang, Cho Lee Wong

**Affiliations:** 1 The Nethersole School of Nursing, Faculty of Medicine, The Chinese University of Hong Kong, Hong Kong, China; 2 School of Nursing, Fudan University, Shanghai, China; 3 Haematology & Oncology Unit, Guangzhou Women and Children’s Medical Centre, Guangzhou, China; 4 Department of Haematology, Hunan Provincial Children’s Hospital, Changsha, China

**Keywords:** Pediatric, Cancer, Chemotherapy, Stress, Coping, Qualitative research

## Abstract

**Background:**

Children undergoing chemotherapy experience significant psychosocial stress; however, limited research has examined how cultural and relational contexts shape stress appraisal and coping within pediatric oncology services in China. Understanding these mechanisms is essential for developing culturally responsive psychosocial care models.

**Methods:**

A qualitative descriptive study was conducted using individual semi-structured interviews with 22 children aged 7–14 years undergoing chemotherapy for haematological cancers in a tertiary children’s hospital in China. Data were analysed using thematic analysis following Braun and Clarke’s framework. Reporting adhered to the Standards for Reporting Qualitative Research (SRQR).

**Results:**

Children’s stress during chemotherapy was shaped not only by physical discomfort and emotional distress, but also by concerns about family burden, disrupted schooling, and loss of normality in everyday life. Children coped mainly through emotion-focused strategies, including holding emotions in, engaging in creative activities, and reframing illness in more manageable terms. These coping practices were influenced by developmental stage as well as cultural and relational contexts, particularly expectations around endurance, emotional restraint, and maintaining stability in family relationships. Coping was also supported through interactions with family members, peers, and healthcare professionals, who provided emotional reassurance, connection, and a sense of safety throughout treatment.

**Conclusions:**

Coping among Chinese children undergoing chemotherapy was shaped by cultural and relational contexts, with important implications for pediatric oncology service delivery. Psychosocial care should move beyond symptom management to include culturally sensitive assessment of family- and school-related stress, support for emotional expression, guidance for caregivers, and opportunities for creative, non-verbal coping within routine care. Strengthening culturally responsive, family-centred services may help support children’s emotional well-being during chemotherapy.

**Supplementary Information:**

The online version contains supplementary material available at 10.1186/s12913-026-14733-6.

## Background

Globally, cancer is a significant cause of morbidity and mortality among children [1]. Haematological cancers account for approximately 30% of the global pediatric cancer burdens and for China [2]. Leukemia and lymphomas are common cancer diagnoses, representing about 70%-75% and 15%-20% of new pediatric haematological cancer cases in China each year, respectively [3, 4]. Beyond their high incidence, haematological malignancies are characterised by prolonged and intensive chemotherapy regimens, high treatment-related toxicity, repeated invasive procedures (e.g., lumbar punctures and bone marrow aspirations), strict infection control measures, and extended or recurrent hospitalisations. Treatment often spans 2–3 years and involves cycles of high-dose chemotherapy [1]. Compared with many solid tumours, this prolonged and uncertain treatment trajectory creates sustained and cumulative stress, disrupting children’s physical well-being, daily routines, and social lives. Such features make haematological cancers a particularly demanding experience for children and highlight the need to better understand how they perceive and respond to illness-related stress.

Children aged 7–14 years represent a substantial proportion of pediatric haematological cancer cases, accounting for approximately 38.5% of diagnoses [4]. This developmental stage encompasses middle childhood and early adolescence, a critical period marked by increasing cognitive sophistication, growing autonomy, heightened peer orientation, and the development of abstract reasoning abilities. During this period, children begin to interpret stressful events more actively, including evaluating threats, anticipating future outcomes, and comparing themselves with others [3]. At the same time, emotional regulation and coping capacities are still developing.

Previous studies show that children with cancer frequently experience significant psychological distress [5], including anxiety, depression, and medical traumatic stress, which may in turn affect treatment adherence, clinical outcomes, and quality of life [6]. Although coping has been widely studied in pediatric oncology, much of the existing research relies on quantitative designs, includes mixed diagnostic groups, or relies on caregiver reports [7, 8]. These approaches may overlook children’s own perspectives and the specific challenges associated with prolonged chemotherapy in haematological cancers. In addition, coping is not only an individual process but is shaped by broader cultural and relational contexts [9, 10]. In Chinese settings, values such as family interdependence, filial responsibility, and emotional restraint may influence how children appraise stress, express distress, and engage with support during treatment [11, 12]. However, there is limited qualitative research exploring how these cultural factors shape children’s stress appraisal and coping experiences within pediatric oncology care in China.

Guided by Lazarus and Folkman’s stress and coping theory [13], this study aims to explore how children aged 7–14 years undergoing chemotherapy for haematological cancers in China perceive and cope with illness-related stress, with particular attention to the cultural and relational contexts in which these processes occur. By adopting a qualitative approach, the study seeks to capture children’s own perspectives on stress appraisal and coping during treatment. A clearer understanding of these processes may inform the development of culturally responsive, child-centred psychosocial support within pediatric oncology services.

## Methods

### Study design

A descriptive qualitative design was applied to capture rich, detailed accounts of participants’ experiences and perspectives [14].

This study was guided by Lazarus and Folkman’s stress and coping theory, which frames stress as a transactional process involving cognitive appraisal and coping responses [13]. The theory guided the development of the interview guide and informed the interpretation of the findings, but it was not used to generate predetermined coding categories. Data were analysed using thematic analysis. The Standards for Reporting Qualitative Research (SRQR) were applied to report this study [15].

### Setting and participants

The study was conducted between July and August 2024 in the pediatric haematology-oncology unit of a tertiary children’s hospital in mainland China. Approximately 1,000 newly diagnosed patients are admitted to this hospital every year. During the data collection period, more patients met the inclusion criteria than were ultimately interviewed. Participants were recruited using purposive sampling with a maximum variation strategy to capture diverse perspectives across age (7–14 years), gender, diagnosis (e.g., leukaemia, lymphoma), and treatment stage [16].

Recruitment occurred during inpatient admissions or scheduled chemotherapy visits. The sample size was guided by the principle of data saturation, defined as the point at which no new themes or substantive insights emerged from subsequent interviews [17]. Based on qualitative research recommendations, we initially anticipated recruiting approximately 20 participants [18]. Recruitment and analysis occurred concurrently. After 20 interviews, preliminary analysis indicated that the themes were well-developed; two additional interviews were conducted to confirm saturation [17]. As no new codes or themes emerged, recruitment was concluded at 22 participants. Thus, the final sample size was determined by saturation rather than by the number of available patients.

A head nurse initially screened participants for eligibility using medical records. To minimise perceived coercion, the nurse introduced the study briefly and asked whether families were willing to receive further information. The principal investigator then contacted interested families, provided detailed study information, and obtained written informed consent from parents or guardians and assent from children.

The inclusion criteria were: (1) aged 7–14 years; (2) diagnosed with a haematological malignancy; (3) currently receiving chemotherapy; (4) able to communicate in Chinese; (5) physically and emotionally able to participate in an interview, as judged by the attending physician; and (6) aware of their diagnosis and treatment situation at a level considered appropriate by their parents and clinical team. Children were excluded if: (1) they or their guardians declined participation; or (2) they had another serious medical condition or psychiatric disorder that would make participation inappropriate. No incentives were offered for participation.

### Data collection

Semi-structured, face-to-face interviews were conducted by the first author, who has extensive experience in qualitative interviewing and pediatric cancer care.

Interviews were conducted in a private room within the hospital to ensure confidentiality and minimise external interruptions. Parents were not present unless the child requested it. When parents remained nearby, they were instructed not to participate in the discussion to preserve the child’s independent voice.

Each interview lasted approximately 30 min (median: 22 min) and was audio-recorded with participant assent and parental consent. Interviews were conducted in Mandarin Chinese and transcribed verbatim. Transcripts were checked against audio recordings for accuracy prior to analysis.

Field notes were taken during and immediately after interviews to capture non-verbal cues (e.g., pauses, tone changes, gestures) and contextual observations.

A semi-structured interview guide was developed by the research team for this study, based on the study objectives, relevant literature [19–21], and stress and coping theory [13]. Given that participants were children aged 7–14 years with haematological cancers, the questions were phrased in age-appropriate, child-friendly language to ensure comprehension and comfort. Abstract theoretical concepts (e.g., appraisal and coping) were translated into simple, concrete questions about children’s thoughts, feelings, and behaviours during illness and treatment.

The guide was pilot tested with two pediatric patients to assess clarity and appropriateness across age groups; no substantive revisions were required.

The full English version of the interview guide is provided as Supplementary File 1.

### Data analysis

NVivo 15 (Lumivero company) was used to manage and organise data. Interviews were transcribed verbatim and analyzed using thematic analysis, following Braun and Clarke’s six-phase framework [22, 23]. First, transcripts were read repeatedly to support familiarisation with the data. Second, initial codes were generated systematically across the dataset. Third, related codes were grouped into potential themes. Fourth, candidate themes were reviewed against both coded extracts and the full dataset to assess coherence and distinction. Fifth, themes were refined and clearly defined. Finally, themes were named and illustrated with representative quotations. Analysis combined inductive coding with theoretically informed interpretation. Initial codes were generated inductively from the data before being interpreted in relation to stress appraisal and coping constructs.

The first author and a research assistant completed transcription. Two researchers independently coded the first five transcripts and discussed their coding to develop an initial coding framework. This framework was revised iteratively as analysis progressed and was applied flexibly to subsequent transcripts. Regular meetings were held to compare interpretations, resolve differences, and refine themes. Where disagreement could not be resolved through discussion, a third senior researcher was consulted.

Negative case analysis was used to strengthen analytic rigour. During theme development, the researchers actively sought accounts that did not fit the emerging patterns, including those of children whose coping responses differed from dominant trends or whose experiences complicated initial interpretations. These cases were revisited alongside the wider dataset and used to refine theme boundaries and wording where necessary.

Throughout the analysis, the team engaged in reflexive discussion about how their disciplinary backgrounds, clinical experience, and cultural positioning might shape interpretation.

### Rigour and reflexivity

Trustworthiness was established through strategies aligned with Lincoln and Guba’s criteria [24]. Credibility was enhanced through peer debriefing and triangulation of interview transcripts with field notes. The first author, who conducted the interviews, is a PhD candidate and registered nurse. The research team remained aware that this clinical background could shape data collection and interpretation, and therefore engaged in ongoing reflexive discussion and bracketing to keep the analysis grounded in participants’ accounts rather than prior assumptions [25]. Dependability was supported through a detailed audit trail documenting research decisions and coding processes. Transferability was addressed by providing rich description of the participants, setting, and context, enabling readers to judge the relevance of the findings to other settings. Confirmability was strengthened through reflexivity and negative case analysis, which were used to question and refine emerging interpretations.

### Ethical considerations

This study was conducted in accordance with the ethical principles outlined in the Declaration of Helsinki. Ethical approval was obtained from the Institutional Review Board of The Chinese University of Hong Kong (Approval No. P84709870). Written informed consent was obtained from parents or legal guardians, and assent was obtained from all participants prior to data collection. Participants were informed of the voluntary nature of participation, assured of confidentiality, and advised of their right to withdraw from the study at any time without any negative consequences.

To protect participants’ privacy, all identifiable information was removed from interview transcripts, and pseudonyms (P1, P2, P3, etc.) were used to represent participants. Given the vulnerability of children undergoing chemotherapy and the sensitive nature of the study topic, the principal investigator—who is a qualified psychological counsellor—was present during interviews and able to provide psychological support if distress arose. Participants and their parents were also informed that they could contact the principal investigator or a hospital-based child psychologist for additional support if needed.

## Results

Sixty patient cases were reviewed, of which 24 met the eligibility criteria. After initial contact, two children declined to participate in the interviews due to experiencing pain from constipation and feeling uncomfortable. Ultimately, this study conducted interviews with 22 (92%) participants. Table 1 presents the characteristics of the participants, who had a mean age of 9.83 ± SD (standard deviation) of 2.53, ranging from 7 to 14 years old. Nine males and 13 females diagnosed with acute myeloid leukaemia (*n* = 18), acute lymphocytic (*n* = 3), and non-Hodgkin’s lymphomas (*n* = 1).

**Table 1:** Characteristics of the participants

Number	Age	Sex	Type of disease
P1	13	male	ALL
P2	7	female	ALL
P3	7	female	ALL
P4	8	female	ALL
P5	8	male	ALL
P6	9	male	ALL
P7	11	female	ALL
P8	9	female	AML
P9	10	female	ALL
P10	9	female	ALL
P11	14	male	ALL
P12	8	male	AML
P13	12	female	ALL
P14	14	male	ALL
P15	14	female	ALL
P16	8	female	ALL
P17	7	female	ALL
P18	7	male	ALL
P19	8	female	ALL
P20	8	female	ALL
P21	11	male	AML
P22	14	male	NHL

NHL: non-Hodgkin’s lymphomas;

ALL: acute lymphocytic;

AML: acute myeloid leukaemia

The data revealed three main themes and ten subthemes (Table 2). These themes explore what is perceived as stress for pediatric haematological cancer patients undergoing chemotherapy and how they cope with stress.

**Table 2:** Themes and sub-themes

Themes	Sub-themes
Culturally Shaped Stress Appraisal in the Context of Illness	• Interpreting Physical and Emotional Suffering • Perceived family burden and responsibility • Disruptions to schooling and social engagement
Culturally patterned ways of coping	• Holding emotions in • Finding comfort in creative activity • Rethinking illness in more manageable terms
Relational support in children’s coping	• Family as emotional grounding and shared responsibility • Friendship as a connection and a sense of normal life • Healthcare professionals as sources of reassurance and emotional safety

### Theme 1: Culturally shaped stress appraisal in the context of illness

Children with haematological cancers who receive chemotherapy not only experience physical or mental stress; they evaluate these challenges through culturally embedded perspectives, intertwining physical discomfort, emotional distress, family expectations and interruptions in daily life. Their perception of stress is largely influenced by family obligations and the cultural expectation to maintain harmony and fulfil social roles in the event of illness. This theme illustrates that children’s stress appraisal processes were not merely a reaction to illness but were also shaped by cultural values around family duty, identity, and societal expectations.

#### Subtheme 1: Interpreting physical and emotional suffering

Chemotherapy-induced physical suffering and emotional distress were interpreted through children’s social and cultural understandings of illness. Children did not describe these experiences solely as bodily discomfort; rather, they linked them to disruptions in normal functioning, peer belonging, and their ability to meet expectations in family and school life.

Many children described chemotherapy as an overwhelming physical experience that altered their sense of self and everyday capacity. One child explained, “When I started the infusion, I didn’t feel as energetic as before” (P5), while another said, “Your body doesn’t adjust well… it’s very uncomfortable” (P22). These accounts suggest that physical suffering was experienced not only as pain or weakness, but also as a loss of normality in a context where children were expected to remain engaged in daily, family, and school routines.

Emotional suffering was further intensified by a cultural context that implicitly discouraged open expressions of distress. Several children described being encouraged by parents to “be brave” and endure treatment without complaint, leading them to suppress expressions of fear and discomfort. This pattern reflects a culturally valued form of emotional restraint, shaping how distress was experienced and managed. Visible physical changes further deepened this distress. One child explained, “I lost my hair. I feel very strange” (P14). In this sociocultural context, where fitting in with peers and maintaining an ordinary appearance may carry social significance, hair loss heightened children’s sense of difference and undermined their sense of normalcy, identity, and belonging.

### Subtheme 2: Perceived family burden and responsibility

Children often understood their illness in relation to its impact on the family. Rather than seeing treatment as something happening only to them, many were acutely aware of the financial pressure, changes in caregiving, and emotional strain it placed on those around them. In this way, stress was closely tied to a sense of responsibility toward the family.

For some, the cost of treatment was itself a source of distress. One child said, “Because of this disease, we’ve spent over ¥200,000 on treatment, and there’s still ¥50,000–60,000 to pay” (P5). This awareness did more than reflect knowledge of household finances; it showed how children were taking in the weight of the family’s situation and, in some cases, quietly carrying guilt alongside their illness.

Children were also sensitive to the ways illness rearranged family life. One participant shared, “After contracting this sickness, my parents travelled far away to seek employment, and my grandfather remained at the hospital with me” (P17). Such changes in caregiving seemed to deepen children’s distress, not only because familiar routines were disrupted, but because illness was experienced as something that had altered the family’s usual ways of being together and caring for one another.

In some cases, this sense of burden became deeply personal. One child simply said, “Mom abandoned me” (P8). Although the circumstances around this experience were shaped by severe family strain, the child appeared to absorb the loss in intensely personal terms. This illustrates how family disruption could be felt not only as sadness or separation, but also as a painful form of self-blame.

#### Subtheme 3: Disruptions to schooling and social engagement

Interruptions to school and everyday peer life were another important part of how children experienced stress. For many, being unable to attend school consistently was not simply an educational problem; it carried emotional weight because school achievement was closely tied to family hopes and to their own sense of doing well as a child.

One participant explained, “I want to study hard and get good grades so that my parents can feel joy” (P5). This comment suggests that academic effort was understood not only in personal terms, but also as something relational, bound up with bringing pride or reassurance to parents. When chemotherapy led to prolonged absence, fatigue, or difficulty concentrating, children were left managing not only illness, but also the worry of falling behind.

Treatment also limited opportunities for play and social interaction. As one child put it, “I can’t play other games with others” (P13). Missing out on ordinary activities with peers appeared to heighten feelings of separation and difference, adding to the sense that illness had pushed them out of the everyday world of childhood.

### Theme 2: Culturally patterned ways of coping

Children described a range of largely emotion-focused ways of coping with the stress of chemotherapy. These responses were shaped not only by age and temperament, but also by the family and cultural environments in which children came to understand illness. In many accounts, coping did not take the form of open emotional disclosure. Instead, children often managed distress through restraint, indirect expression, and efforts to reinterpret illness in more bearable terms. Taken together, these patterns suggest that coping was shaped by expectations of endurance, emotional control, and the preservation of relationship stability.

#### Subtheme 1: Holding emotions in

Some children coped by keeping difficult feelings to themselves. In several accounts, opportunities to speak openly about fear, sadness, or frustration seemed limited, and children described wanting more encouragement from parents to express what they were feeling. As one participant said, “I hope my parents encourage us more to express ourselves” (P18). Other accounts suggested that children were often encouraged to be brave and endure treatment without complaint. In this setting, emotional control appeared to be valued, and some children seemed to respond by containing their distress rather than voicing it directly. Coping therefore often took the form of endurance and self-restraint, with children managing distress inwardly within a family atmosphere where being strong was quietly encouraged.

#### Subtheme 2: Finding comfort in creative activity

Many children found comfort in quiet, creative activities such as painting, handcrafting, reading, singing, or simply looking out at the view. For these children, such activities offered more than distraction. They created space for calm, relief, and a form of expression that did not rely on speaking openly about difficult feelings. Painting and handcrafting were especially meaningful in this regard, as they seemed to give children a way to express emotion indirectly and in manageable ways. One child explained, “Before I was ill, I preferred dancing. Chemotherapy makes me weak. I began liking painting and handmaking to express myself” (P7). For children whose energy was limited and whose emotions were not always easy to express, these quieter activities appeared to offer a culturally and developmentally acceptable way to cope.

#### Subtheme 3: Rethinking illness in more manageable terms

A small number of older children described coping by deliberately changing how they thought about their illness. Rather than allowing cancer to dominate their sense of self, they tried to place it within a broader life perspective, seeing it as a difficulty to get through rather than something that defined them. As P11 said, “Actually, after adjusting mindset, you won’t think of this disease as so serious…” Similarly, P14 reflected, “Later, I thought this was just a difficulty in my life. We should look at it from a positive perspective.” This form of cognitive reframing was more evident among the older participants and suggests a developmental shift toward more reflective, meaning-based coping. For these children, coping seemed to involve preserving hope and emotional steadiness by making illness feel less overwhelming and more containable within the larger course of life.

### Theme 3: Relational support in children’s coping

Children did not cope with chemotherapy alone. Across the interviews, relationships with family members, peers, and healthcare professionals were described as central to how children managed fear, discomfort, and uncertainty during chemotherapy. These forms of support went beyond practical help. They shaped how children made sense of their experience, helped regulate distress, and provided a sense of stability during a period of disruption. In this context, coping was not simply an individual effort, but something sustained within a network of relationships that carried emotional and, at times, cultural meaning.

#### Subtheme 1: Family as emotional grounding and shared responsibility

Family support was woven through nearly all accounts. Children described parents, grandparents, and siblings as those who stayed close, offered reassurance, and helped them get through difficult moments. This support was both practical and emotional. For some, simple words of encouragement helped reduce fear and made treatment feel more manageable. One child recalled, “Mum said I need to relax to get well quickly” (P13).

At the same time, family support often reflected a shared sense of responsibility, where illness was experienced not just as an individual condition but as something the whole family carried together. When parents were absent due to work or financial pressures, other relatives stepped in. As one participant said, “My grandmother takes good care of me, especially when I feel uncomfortable during infusion” (P1). In these situations, care from family members seemed to provide emotional grounding, helping children feel looked after and less alone, even when circumstances were difficult.

#### Subtheme 2: Friendship as a connection and a sense of normal life

Friendships offered a different kind of support. For many children, friends were a source of comfort, encouragement, and continuity with life outside illness. One child explained, “For such a long time to receive the chemotherapy, the comfort from friends has also kept me going” (P15). These relationships appeared to help sustain emotional strength over time, particularly during prolonged treatment.

Children also spoke about wanting to make new friends in the hospital. “I want to make new friends here” (P19), one participant said. Forming connections with peers in similar situations seemed to reduce feelings of isolation and create a sense of shared understanding. In this way, friendships helped children maintain a sense of belonging and normality, even in the unfamiliar hospital environment.

#### Subtheme 3: Healthcare professionals as sources of reassurance and emotional safety

Healthcare professionals were also described as important in helping children cope, not only through medical care, but through everyday interactions that made the hospital feel less intimidating. Children often spoke about doctors and nurses in relational terms, noting their kindness, humour, and attentiveness. One child recalled, “Those doctors and nurses are all very nice. One time, when the nurse sisters visited to check in, they discovered I was miserable and even joked with me” (P11).

These moments, although small, appeared to ease emotional tension and make children feel more comfortable during treatment. In addition, hospital-based activities created opportunities for children to engage, interact, and express themselves more freely. As one participant noted, “After having taken part in the activities organised by the hospital, I was more ready to speak” (P13). This suggests that support from healthcare professionals extended beyond clinical tasks, creating an environment in which children felt safer, more at ease, and more willing to communicate.

## Discussion

This study offers insight into how Chinese children undergoing chemotherapy for haematological cancers perceive and manage illness-related stress within pediatric oncology care. Overall, the findings suggest that children’s coping was shaped not only by the physical demands of treatment but also by the cultural and relational contexts in which illness was experienced. Children did not speak about stress as a purely bodily or individual burden. Rather, they interpreted it through concerns about family burden, school disruption, emotional restraint, and their place within close relationships. In this sense, coping was not simply a matter of developmental capacity or personal temperament; it was also shaped by family expectations, caregiving practices, and interactions with healthcare professionals. By bringing children’s own accounts to the foreground, this study adds to pediatric oncology literature by showing that stress appraisal and coping are embedded in social and moral worlds that matter deeply to children.

### Culturally shaped stress appraisal

A key finding of this study is that children’s stress during chemotherapy was not limited to physical suffering but was closely tied to how illness disrupted valued roles and relationships. Physical discomfort, fatigue, pain, and treatment side effects have been widely reported in pediatric oncology research [26, 27]. However, our findings suggest that children did not experience these symptoms only as bodily distress. They also understood them as affecting their ability to participate in school, maintain peer relationships, and respond to family expectations. This is important because it suggests that stress was appraised not only in medical terms, but also in relation to what children felt they ought to be able to do in everyday life.

Academic disruption was especially notable in this regard. Previous studies often discuss school absence as a practical or developmental challenge [28]. In this study, however, children’s concerns about school were closely tied to family meaning. Academic achievement was not described simply as personal success, but as something linked to parental hopes and family pride. In this context, treatment-related disruption could be experienced as more than an interruption; it could take on moral and relational significance. This finding helps explain why school-related stress figured so strongly in children’s accounts and suggests that educational disruption may carry a different emotional weight in settings where academic achievement is strongly embedded in family expectations.

The findings also point to the central place of family burden in children’s appraisal of illness. Many participants were aware of treatment costs, changing caregiving arrangements, and parental strain [29, 30]. Some appeared to interpret their illness in ways shaped by family responsibilities and indebtedness, echoing cultural values of interdependence and filial concern [31, 32]. Rather than expressing distress openly, some children seemed to contain their feelings to avoid adding to parental worry. This suggests that stress was not experienced only as something happening to the child, but also as something understood through its perceived effects on the family. Recognising this relational dimension is important because it shifts attention from symptom burden alone to the moral and emotional meanings children attach to illness within family life. Children’s concerns about family burden and school disruption also suggest the need for psychosocial assessment that extends beyond symptom distress to include relational guilt and educational uncertainty.

### Coping as culturally and developmentally shaped

The second important finding concerns the ways children coped with chemotherapy-related stress. Consistent with stress and coping theory [13], children relied largely on emotion-focused coping strategies in response to stressors that were often beyond their control. However, these strategies were not simply individual responses to illness. They appeared to be shaped by both developmental stage and the cultural and relational contexts in which children learned to manage distress [33, 34].

One of the clearest patterns was children’s tendency to hold in their emotions. Some participants described limited encouragement to speak openly about sadness, fear, or frustration, and several accounts suggested that being “brave” and enduring treatment quietly was implicitly valued. In this context, emotional restraint may have served to protect relationships and preserve family stability, rather than as a sign of emotional resilience alone [35, 36]. This is an important distinction. If emotional suppression is read only as individual temperament or strength, there is a risk that unmet emotional needs will remain unnoticed. Our findings suggest instead that silence may sometimes reflect a relationally shaped coping pattern, formed within caregiving environments where emotional expression is not always actively invited.

At the same time, children also described more adaptive ways of managing distress, particularly through creative activity and cognitive reframing. Drawing, handmaking, and similar activities offered more than a distraction. For some children, they appeared to provide an indirect, manageable way to express emotion when direct verbal disclosure felt difficult. This may be particularly relevant in contexts where emotional restraint is common and non-verbal channels of expression are more acceptable or more comfortable [37–39]. Such activities enabled children to regain a sense of agency and achievement despite physical limitations. Rather than treating creative activity as peripheral or recreational, these findings suggest it may function as a meaningful coping practice. This suggests that children who cope through emotional restraint may benefit from routine, developmentally appropriate opportunities for emotional expression, including non-verbal and creative forms of support.

Cognitive reframing was more evident among the older children in the sample, who were sometimes able to place illness within a broader life perspective and view treatment as something difficult but temporary. This pattern is consistent with developmental research suggesting that older children and young adolescents may have a greater capacity for reflective and meaning-based coping [33, 34]. Together, these findings indicate that coping during chemotherapy is shaped by an interplay of developmental capacity, relational environment, and cultural expectations around emotional control and endurance.

### Contribution to pediatric oncology service research

This research has contributed to pediatric oncology in several ways. First, it shows that children’s stress during chemotherapy is not only driven by symptoms, but also explained by family roles, school expectations and relationship concerns. This broadens the current understanding of psychosocial needs, indicating that children may suffer not only from the disease itself, but also from what the disease means in the social world in which they live.

Secondly, research has found that coping behaviour is often influenced by cultural expectations and the nursing environment. For example, emotional suppression should not be regarded as merely a personal way of coping, but as something that may occur in the family environment. Endurance and emotional control are quietly valued in the family setting. This highlights the importance of supporting care workers in psychosocial care, not only isolated children.

Third, the research results have attracted people’s attention to the positive role of medical staff in children’s emotional regulation. From the child’s point of view, the staff is not only a provider of treatment, but also a contributor to assurance, trust and emotional comfort. This enhances the relevance of communication, relationship continuity and daily emotional support in pediatric oncology services.

### Practice implications

This study underscores the importance of embedding culturally responsive psychosocial care within pediatric oncology service delivery. The findings suggest that children’s stress extends beyond physical symptoms and is shaped by perceived family burden, school disruption, and culturally influenced emotional restraint. Routine psychosocial assessment should therefore move beyond symptom-focused screening to include children’s concerns about relational guilt, academic disruption, and indirect expressions of distress. Structured emotional check-ins and developmentally appropriate, non-verbal communication strategies may help identify unmet psychosocial needs earlier.

The findings also point to the importance of supporting caregivers. Parents may benefit from guidance on recognising children’s emotional needs, inviting expression without pressure, and responding in ways that offer both reassurance and openness. This may be particularly important in contexts where children are encouraged to be strong, and distress is not always voiced directly. Interdisciplinary collaboration is also needed. Nurses, physicians, psychologists, social workers, and child-life specialists should work together to integrate psychosocial support throughout the treatment trajectory rather than offering it only in response to a crisis.

At the service level, creative and non-verbal forms of psychosocial support should be considered part of routine supportive care rather than optional recreational activities. Art-based and other expressive approaches may provide culturally and developmentally appropriate ways for children to communicate distress when verbal expression is limited. In addition, strengthening family-centred counselling and providing access to practical support, including financial guidance where needed, may help reduce the relational pressures that intensify children’s distress. Embedding these approaches within routine pediatric oncology care may help create more responsive and emotionally supportive services for children undergoing chemotherapy.

### Limitations

This study has several limitations. First, participants were recruited from a single hospital and limited to children with haematological cancers, which may restrict the transferability of findings; future studies should use multi-site sampling across diverse settings and cancer types. Second, this qualitative study captured children’s coping experiences at a single point during chemotherapy, it may not fully show how coping strategies change over the treatment trajectory. Future longitudinal research could examine how culturally shaped coping evolves across treatment phases and how pediatric oncology services can respond to children’s changing psychosocial needs.

Third, the study relied on children’s self-reports, which may be influenced by social desirability and limited emotional articulation, and the absence of parents’ and healthcare providers’ perspectives limited opportunities for data triangulation.

## Conclusion

This study shows that children undergoing chemotherapy for haematological cancers in China experience and manage stress within cultural and relational contexts, rather than as an individual response to illness alone. Children’s stress was shaped not only by physical suffering but also by concerns about family burden, school disruption, and maintaining emotional control. Coping was supported through creative activities, family relationships, peer connection, and emotionally attuned interactions with healthcare professionals. These findings highlight the need for child-centred, culturally responsive, and family-inclusive psychosocial care within pediatric oncology services. Supporting emotional expression, strengthening family communication, and integrating creative and relational forms of care into routine practice may help improve children’s emotional well-being during treatment.

## Supplementary Information

Below is the link to the electronic supplementary material.

Supplementary Material 1

## Data Availability

The datasets used and analysed in the current study are available from the corresponding authors upon reasonable request.

## Abbreviations

SRQR: Standards for Reporting Qualitative Research
SD: Standard deviation
