# Supplementary material for: Cultural influences on coping among Chinese children aged 7–14 undergoing chemotherapy: implications for pediatric oncology service delivery
Source: BMC Health Serv Res. 2026 May 23;26:1002. doi: 10.1186/s12913-026-14733-6 (PMC13386690; doi:10.1186/s12913-026-14733-6)
Supplement: Supplementary file 1 — Supplementary Material 1 [file 12913_2026_14733_MOESM1_ESM.docx]

**Supplementary File 1**

**Semi-Structured Interview Guide**

**Introduction**

Hi, thank you for talking with me today.

I would like to learn about your experiences with your illness and treatment. There are no right or wrong answers. You can choose not to answer any question, and you can stop at any time.

**Main Questions**

1. Can you tell me what you think about the illness you have?

2. What things during treatment or chemotherapy make you feel worried, upset, or stressed?

3. When you feel that way, what do you usually do?

4. Is there anyone or anything that helps you feel better?

5. Is there anything else about your experience that you would like to share?

**Possible Prompts**

• Can you tell me more about that?

• How did that make you feel?

• What happened next?

• What did you do then?

• Has this happened before?
